# Exploring the Asthma Network in People with Allergic Rhinitis Utilizing an Egocentric Social Network Analysis

**DOI:** 10.1007/s41030-019-0095-9

**Published:** 2019-07-12

**Authors:** Biljana Cvetkovski, Rachel Tan, Vicky Kritikos, Kwok Yan, Elizabeth Azzi, Pamela Srour, Sinthia Bosnic-Anticevich

**Affiliations:** 1 Woolcock Institute of Medical Research, The University of Sydney, 431 Glebe Point Rd, Glebe, Sydney, NSW 2037 Australia; 2 Royal Prince Alfred Hospital, Camperdown, Sydney, Australia; 3 Sydney Local Health District, Sydney, Australia

**Keywords:** Asthma, Allergic rhinitis, Healthcare professionals, Patient

## Abstract

**Introduction:**

Asthma and allergic rhinitis (AR) are chronic respiratory diseases of a united airway. Poor AR control is a risk factor for uncontrolled asthma. We know that people with AR feel confident in making their own treatment choices with over-the-counter therapies, yet only 16% of purchases were the optimal selection. With the high level of poor asthma control and overuse of over-the-counter, short-acting beta-agonists, we must consider whether poor AR self-management behaviours are extended to asthma management in those with both diseases. This study aims to investigate asthma management from the perspective of the patient with asthma and AR and understand the influences behind their asthma management decisions.

**Methods:**

This study utilized a mixed methods approach based on the theoretical and analytical framework of social network theory, including mapping of the asthma network and exploring the roles and influence of those that appear within the network.

**Results:**

Twenty-two people with asthma and allergic rhinitis participated in this study. General practitioners (GPs), pharmacists and respiratory physicians were the most commonly reported influences behind participants’ asthma management decisions. Although non-healthcare professional (HCP) influences appear within the asthma network, they represented a smaller proportion.

**Conclusion:**

The asthma network of people with AR is dominated by HCP influences. This network is unique and different to other previously published asthma and AR networks. Further research on the impact of AR on asthma management patient behaviour is required.

**Electronic Supplementary Material:**

The online version of this article (10.1007/s41030-019-0095-9) contains supplementary material, which is available to authorized users.

## Introduction

Asthma and allergic rhinitis (AR) are chronic respiratory diseases of a united airway [1–4] that present a significant clinical and socioeconomic burden on society, ranging from increased utilization of health resources to impairment in workplace productivity [5–10]. In addition to burdens they impose on people independently, AR is an independent risk factor for the development of asthma [11] and as a co-morbidity of asthma is associated with impaired asthma control, reduced asthma-related quality of life and a factor in the overutilization of asthma-related resources [12, 13]. The effect of AR in people with asthma cannot be ignored with 78% of people with asthma having AR [14] and its association with tragically fatal ‘thunderstorm asthma’ in Australia, where 87% of patients requiring hospital admission had rhinitis [15, 16].

Although asthma management guidelines recommend identifying and treating co-morbid allergic rhinitis, this is a challenge in practice because of the high rates of underreporting and underdiagnosis of AR [17–19]. A fervent example of this is an Australian cross-sectional study conducted by Bosnic-Anticevich et al. [20] which comprehensively reviewed 200 people with asthma within the general practice setting. Ninety per cent of the patients reviewed reported nasal symptoms, yet only 45.6% had received a diagnosis and 67.3% of the people with moderate–severe rhinitis were not using the recommended intranasal corticosteroid (INCS) therapy [20]. If we are to improve asthma control, we must understand why such a large proportion of people do not report and treat their nasal symptoms while they are seeking asthma treatment.

Investigations into the patient perspectives of allergic rhinitis management in the Australian setting have provided some insight into why some nasal symptoms continue to be suboptimally controlled. Patients have reported incidences of delayed diagnosis, treatment fatigue and confidence in their ability to manage their condition themselves with over-the-counter (OTC) therapies. Despite this confidence, only 16.5% of people self-selecting medicines in a pharmacy for their AR make an optimal selection [18]. Social network research of AR management from the patient’s perspective further details the extent of influence of patient-driven self-management. Social network investigations of key influences on patient’s AR decision-making demonstrated that the patients ‘own experience’ was the third most influential factor, behind advice from a general practitioner (GP) and pharmacist [21]. When considering these findings, one considers whether this prominent patient confidence in their ability to self-manage their own condition is specific to AR or common to AR and asthma in people with both co-morbidities.

Cheong et al. utilized social network theory to identify the asthma network in two Australian populations of asthma patients, those that primarily consult a GP and those that consult a specialist for their asthma management [22]. These networks explored with whom the patients discuss their asthma and demonstrated a strong presence of non-healthcare-trained family and friends in addition to healthcare professionals (HCPs). However, Cheong and colleagues did not distinguish people with AR among the study population. We need to understand whether AR management behaviour is related to asthma management behaviour and whether people with AR are more or less likely to self-manage their asthma. This is especially crucial in the Australian setting which has a high level of poor asthma control and overuse of short-acting beta-agonists which are available for purchase without a doctor’s prescription.

To investigate whether patient asthma management behaviour is influenced by having AR, we can start by mapping the asthma health network for people with asthma and AR. We hypothesize that the map will be different to the asthma map defined by Cheong et al. and that some of the influences identified in the AR network [21] will appear within the asthma map of those with AR.

This research aims to map the asthma network of people with asthma and AR and explore the relationships within it.

## Methods

### Study Design

This study used a mixed methods approach based on the theoretical and analytical framework of social network theory as previously developed and utilized by Cheong et al. [22] and Cvetkovski et al. [21]. Specifically, this study used an egocentric social network framework; it focused on the network of an individual/‘ego’ (the participant) and the relationships/‘ties’ with individuals or resources/‘alters’.

We addressed the aim of the study by (1) drawing the health networks of people with asthma with co-morbid AR and identifying alters within the network, (2) determining the degree of influence of each alter within the network and (3) exploring the participants’ perceptions of the roles of alters within the network.

### Study Population

#### Inclusion Criteria

The target population was people aged 18 years or older who identified themselves as having AR and asthma, had consulted a doctor for their asthma and were able to speak English.

#### Recruitment

A convenience sample of people who had recently participated in a study at the Woolcock Institute about their AR network [21] were asked if they also had asthma and invited to participate in this study. Written consent was obtained from participants prior to commencement in the study.

#### Sample Size

All people within the group that identified themselves as having AR and asthma were included in the study.

### Data Collection

All data was collected through face-to-face or telephone interviews, depending on the participant’s preference. Participants who chose to be interviewed over the telephone were posted/emailed the supporting documents and questionnaires, to have in front of them for reference during the telephone interview. All interviews were audio recorded and transcribed verbatim.

In order to achieve the aim of this study, the interview process consisted of three stages/phases, during which three processes were followed:

1. Asthma status questionnaires: Asthma control and quality of life were determined by administration of validated questionnaires [23, 24].
2. Semi-structured interview: The semi-structured interview incorporated the name generator technique [25] and the name interpreter technique [26]. Participant were asked questions to help them identify/generate a list of alters (individuals or resources) with whom they have discussed their asthma, with within the last 5 years. A predetermined list of potential alters, generated from asthma literature [8, 27, 28] and exploratory inquiry, was used as secondary prompts in this process. Participants were also required to described the role of the alters with respect to their asthma management and provide specific examples if possible. The interview guide is available as a supplement.
3. Mapping of the asthma health network: An adapted concentric circle framework was used as the basis for the generation of each asthma network map [29]. The participant was asked to visually depict their relationship with each of their alters with respect to their influence on their asthma management, by mapping them on a concentric circle diagram [21]. The red circle in the centre represents the participants and each ring around the centre represents the strength of influence that an alter has on their asthma management, i.e. circle 1 contained alters with the strongest relationship and circle 4 contained the weakest.

### Data Analysis

1. Asthma control and impact on quality of life: Asthma control was evaluated using the 6-item asthma control questionnaire (ACQ) [23]. Asthma-related quality of life was assessed using the mini asthma quality of life questionnaire (mAQLQ) [30]
2. Mapping of asthma networks: The asthma health network data was represented in two ways:
  - Asthma network maps and bar graphs
    The asthma network maps were drawn using NetDraw [31, 32]. An asthma network map collating all participants’ individual maps was drawn. Asthma network bar graphs were drawn to quantitatively depict the asthma network map described above.
  - Asthma network alter density graph
    Network alter density = $\frac{{{\text{alter score}}*}}{{{\text{sum of all alter  scores}}}}$ × 100 $*{\text{Alter score}}\,{\kern 1pt} = \,{\kern 1pt} n_{ 1} C 1\, \times \, 4\, + \,n_{ 2} C 2\, \times \, 3\, + \,n_{ 3} C 3\, \times \, 2\, + \,n_{ 4} C 4\, \times \, 1$ (*C* refers to circle)
3. Alters within the asthma network
  Qualitative social network analysis was utilized to identify descriptions of the roles of alters as described by the participants within the transcripts. A deductive approach was employed where the authors searched the transcripts for significant statements describing the pre-determined themes of ‘role’ and ‘function’ of alters with respect to asthma management and their place within the network.

This study was approved by the University of Sydney Human Research Ethics Committee and was completed in accordance with STROBE guidelines for observational research [33] and COREQ guidelines for qualitative research [34]. This study was performed in accordance with the Helsinki Declaration of 1964 and its later amendments. Informed consent was obtained from the participants to participate and publish a manuscript.

## Results

### Study Population

Twenty-four people volunteered to participate in this study and provided written informed consent, which represented all the people in the original study that identified themselves as having AR and asthma. Two were excluded because they had not consulted a doctor for their asthma and were managing their ‘asthma’ by purchasing OTC salbutamol for shortness of breath. The remaining 22 people were included in the study and signed informed consent. Eighteen participants were female and four were male, aged 22–64 years, with an average age of 48 and a median age of 38. Four participants were from rural New South Wales and 18 from metropolitan Sydney. Data saturation was reached following the 14th participant, after which no new alters were identified within the subsequent asthma maps.

1. Disease status: Asthma control (ACQ) and quality of life questionnaires (mAQLQ) were completed and returned by 20 participants. The participants with outstanding questionnaires were sent a prompt and an additional copy of the questionnaire to complete but it was not received by the researchers. ACQ scores are displayed in Table 1. Mean mAQLQ score (± SD) was 0.953 (± 0.996).

**Table 1:** Asthma control and alters

Asthma control ACQ scores	Number of participants	Average number of alters in network map
Controlled score ≤ 0.75	10 (52%)	4
Partially controlled 0.75 < score < 1.5	2 (38%)	3
Uncontrolled score ≥ 1.5	8 (38%)	5
2. Asthma network: (a) Asthma network map and bar graph (Fig. 1)

**Fig. 1:**
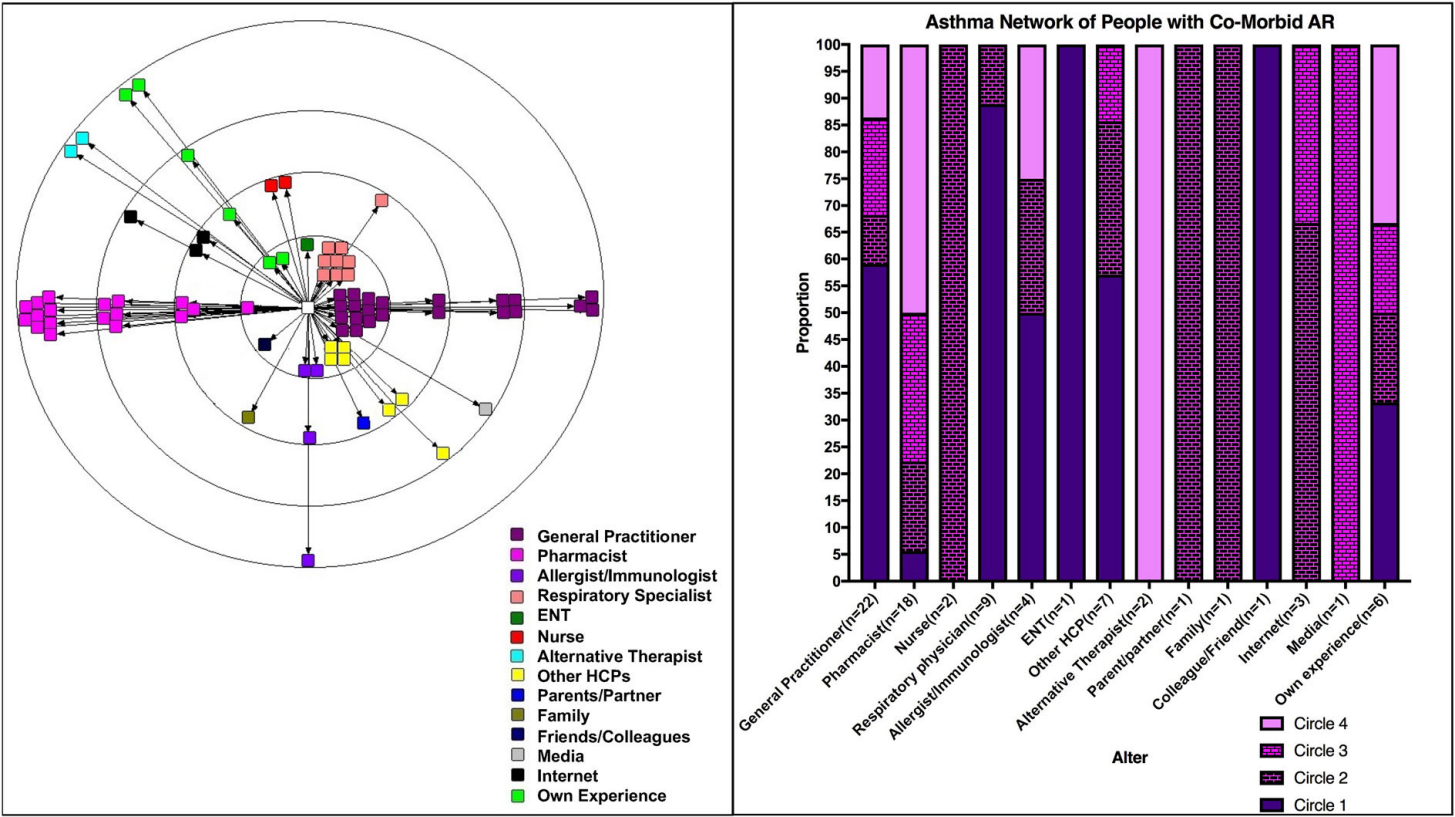
Asthma network map and bar graph

  Average number of alters per network (4), min (1), max (10) and median (3) and mode (3).
    Alter average per asthma control category is displayed in Table 1.
    The type of alters that appeared in the asthma network included GPs, pharmacists, allergists/immunologists, respiratory physicians, ear, nose and throat (ENT) specialists, nurses, alternative therapists, parents/partners, family, friends/colleagues, media, internet, own experience and other HCPs (ambulance officers, dentists, ophthalmologists, physiotherapists and dermatologists).
    GPs were in every participant’s asthma network and placement was throughout all circles in the map, with 55% of participants placing them in circle 1, indicating the strongest influence on their asthma management. Pharmacists were the second most frequently mentioned alter and were placed in every circle in the map but were predominantly placed in circle 4 (50% of pharmacists), indicating the lowest level of influence on asthma management. Respiratory physicians were the third most commonly mentioned alter and were placed in circle 1 in 89% of cases, with one exception of placement in circle 2.
  (b) Asthma network alter density (Fig. 2)
  The asthma network alter density figure (Fig. 2) illustrates that HCPs represent more than 80% of the alter density within the asthma network and are the dominant influence with regards to asthma management from the perspective of the patient. GPs and respiratory physicians alone contribute to almost 50% of the influence within the asthma network.

**Fig. 2:**
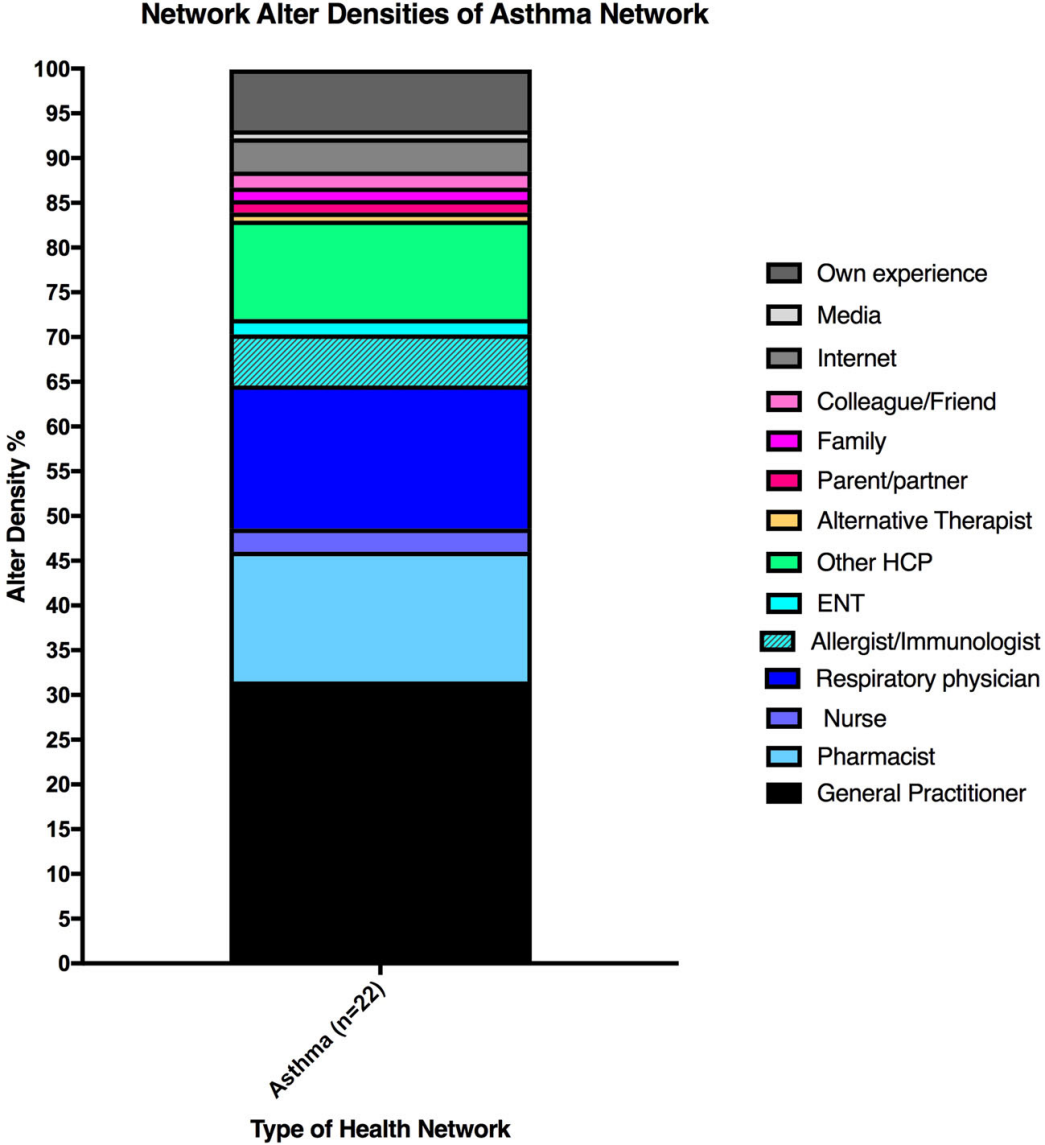
Asthma network alter density
3. Roles of the alters within the asthma network
  As participants nominated each alter, they were asked to describe their role with regards to their asthma management. Descriptions of GP influence with regards to asthma management were varied, similarly to the scattering of placement of GPs within the asthma map. Participants valued their GP for advice on how to manage their asthma symptoms but also valued their practical role such as writing a prescription for asthma medicines, with some participants visiting GPs at medical centres to obtain prescriptions. Participants that placed GPs in circle 4 had had negative experiences with GPs with regards to asthma advice. Participants reported GPs highlighting the association between AR and asthma and alerting them to the impact of AR on their asthma.
  Participants that nominated their own experience within the asthma network placed it within the network map depending on their confidence with making their own decisions with the information available to them. A participant that placed her own experience within circle 1, along side her GP, felt that she adjusts her Symbicort^®^ use when she feels her asthma getting worse.
  Respiratory specialists were highly influential with regards to participants’ asthma management. Some participants reported bypassing their GP and seeking a consultation with the specialist when they felt their asthma getting worse. The only time they were placed outside of circle 1 (circle 2) was because the participant felt the infrequent consultations they had with them did not qualify them for placement within circle 1. Participants reported that consultations with their respiratory specialists focused primarily on their asthma.
  Allergists had been referred to by GPs to identify whether there was an allergic component to their asthma. However, participants were initially unsure of the relevance of allergists to their asthma management.
  The media, including the television news and newspapers, had a role to play with regards to alerting participants to new breakthroughs with regards to asthma. Information pamphlets provided in pharmacies, hospitals and doctors’ surgeries were also mentioned for providing information with regards to management and awareness.
  Participants reported the role of friends and colleagues as one of emotional support as well as a reference source for ascertaining the value of other management strategies.
  Nurses’ roles within the asthma network were with regards to asthma education, asthma review and medication adjustment.
  The patient reported being referred to an ENT for their allergy specialty following identification by the respiratory physician that their AR is making their asthma worse.
  The majority of participants that nominated a pharmacist within the asthma network described their role as one of supply of prescription medication. Other participants had pharmacists that offered inhaler technique education and management advice.
  Participants that had alternative therapists within their network had consulted them for alternatives to medication for managing their asthma symptoms. They were placed within the outer circles because the recommended therapies were not perceived to be effective.
  Participants reported exploring the internet with regards to information about their asthma management and for instruction on how to use their device. Participants were wary of incorrect information and would refer to reputable websites from medical organizations or check with their doctor about the information they had found.
  Physiotherapists were reported to have a diverse role with regards to asthma management. Some participants reported physiotherapists providing general asthma education, others specific inhaler technique instructions as well as therapy for chest clearance. A dermatologist was identified by a participant as having a role within the asthma network for managing the adverse effects associated with oral prednisone use for asthma. The ophthalmologist was identified for monitoring with regards to the manifestations of oral steroid use for asthma on the eyes.
  The ambulance service was nominated for their role in taking the participant to the hospital when their asthma had flared up. Evidential participant quotes are available as a supplement.

## Discussion

This study has identified the asthma network map in people with co-morbid AR and demonstrated that HCPs, led by the GP, represented the majority of the influences with respect to the patient’s asthma decision-making. The roles of the HCPs within the asthma network were identified to be addressing multiple facets of asthma management, including prescriptions and provision of medication, asthma education, monitoring of side effects and inhaler technique education. While non-HCP alters were also within the network, their roles were complementary to that of the HCP and the information obtained from them was often echoed back to the HCP for consideration and discussion.

People with asthma and AR reported a broad range of roles for HCPs in their asthma network. The role of the GP has been further demonstrated as influential in asthma management, regardless of the different roles perceived by the patient. The pharmacist continues to be perceived as a supplier of medication with little influence in asthma management, which was also found by Cheong et al. Respiratory physicians have a strong influence in a patient’s decision-making, often superseding the GP when accessible. The nomination of other HCPs not normally associated with asthma management, such as dermatologists and ophthalmologists, demonstrate that in severe disease and the adverse effects associated with high dose corticosteroids, more body systems are affected and subsequently more HCPs are consulted. The influence of non-HCP people and resources appear less influential than previously reported [35] and offer more of a supportive role rather than that of influence in management decisions.

When contemplating whether AR as a co-morbidity has an impact on the asthma network, we can compare the results of our study to that of Cheong et al. [35]. Cheong et al. mapped the asthma networks of people with asthma that had been recruited in from a general practice (*n* = 26) and that of people recruited from a specialist respiratory clinic (*n* = 21). Both of Cheong et al.’s asthma maps had a strong presence of family and friends, indicating that family and friends were particularly influential in a person’s asthma management decisions. GPs and specialists also featured in both maps; however, specialist influence also superseded GP influence in the group recruited from the specialist clinic. Our sample size (*n* = 22) is comparable to Cheong et al.’s groups but have overall better asthma control with more than 50% reporting an ACQ of less than 0.75 (Cheong et al. reported more than 40% had ACQ less than 1), with fewer male representatives but a similar average age. If we consider comparing our map to Cheong et al.’s map, we see that our maps have a less pronounced nomination of family and friends and nomination of additional HCPs that did not appear in Cheong et al.’s maps. However, our participants also nominated fewer alters within their network. Cheong et al.’s network mentioned the significant role of family and friends in asthma self-management, which was not the case in our asthma network. The asthma networks are quite different between the two studies demonstrating that social network analysis data is a cross-sectional representation of a particular population at a particular moment in time and the results are not necessarily transferrable among similar patient groups. Slight variations in recruitment strategies, introduction of co-morbidities into the discourse and possible variation in researcher techniques during the interview with participants can all possibly contribute to the difference in asthma network maps between the two studies.

In light of the asthma network in people with co-morbid AR being different to previously published asthma networks [35], it is necessary to compare it to the previously published AR network for possible similarities or differences [21]. The AR network map’s study population was twice the size of the one in this study, had more male participants, a larger range of AR severities and more representatives from non-metropolitan areas, which can be contributing factors to differences in the network. Upon inspection, the AR network map has slightly reduced influence from GPs, more influence from pharmacists, more influence from the participants’ own experience and more influence from parents/partners than our asthma map, demonstrating a clear difference in the influences and health behaviours that patients have towards their AR and asthma. This comparison between our asthma network in people with co-morbid AR versus the AR network clearly demonstrates that people treat both conditions differently; however, the influence of differences in demographics and socio-economics in the participant groups cannot be discounted and must be considered in future research.

Upon considering why people with asthma and AR treat their conditions differently, we must also look toward patient perceptions of each disease. Through the media AR is portrayed as a nuisance condition through advertisements for antihistamines and patients feel they have ‘lay expertise’ [36] and are able to manage it themselves. Yet asthma is often brought to light in relation to hospitalisations or tragic deaths [15, 17]. The relationship between AR and asthma was highlighted in the Australian media following a tragic thunderstorm asthma event; however, it is to be questioned how many people outside the medical profession identify the connection between the two diseases. While people with AR have reported the debilitating effects of AR as being far from a nuisance [37, 38], it seems that this message is not broadcast to the general population with some sufferers even choosing not to discuss the debilitating effects with those around them and trying to appear unaffected [39]. Research exploring people’s perception of asthma has demonstrated that they often perceive their control to be better than it is [20] and try to spare their family and friends from the significance of their flare-ups by choosing understated language to describe their exacerbation that will not cause alarm [40]. We also know that a patient’s health beliefs [41] can influence their health management and future research must also take this factor into account.

The small sample size of this study, the limited representation from the male population and those living outside of the Sydney metropolitan area represent a significant limitation of this study. The study further limits the direct comparison of asthma networks between this population and that of Cheong et al. by having variations in data collection with respect to the name generator and name interpreter questions of the semi-structured interview, which were not as in-depth as that of the previous study. Not confirming the participants’ diagnosis of AR and asthma also represents a significant limitation; however, this is representative of the populations that self-manage their conditions with OTC therapies without consulting an HCP. Further, qualitative research with a larger, more diverse study population is required to provide an in-depth understanding of the influence AR self-management has on asthma management behaviours.

In conclusion, the asthma network in people with AR is dominated by HCPs with the GP and respiratory specialist having the most influence on patients’ asthma management decisions. Having asthma and AR has an impact on the asthma network with differences apparent when compared to that of the previously reported asthma network and the AR network; however, further research is required with a larger sample size with comparable demographic and socio-economic profiles. Further exploration into the impact of patient personalities and health beliefs on the asthma network will identify the influences on asthma management decisions for individual patients.

## Electronic supplementary material

Below is the link to the electronic supplementary material.

Supplementary material 1 (PDF 80 kb)
