# Supplementary material for: Exploring the Asthma Network in People with Allergic Rhinitis Utilizing an Egocentric Social Network Analysis
Source: Pulm Ther. 2019 Jul 12;5(2):235–45. doi: 10.1007/s41030-019-0095-9 (PMC6967048; doi:10.1007/s41030-019-0095-9)
Supplement: Supplementary file 1 — Supplementary material 1 (PDF 80 kb) [file 41030_2019_95_MOESM1_ESM.pdf]

**Name Generator Technique**	Tell me about your asthma management and who or what influences your management?	How does a GP, pharmacist, specialist, family, friend, the internet and media influence what you do about your asthma?	Who have you spoken with about your asthma?
**Name interpreter questions**	Tell me about how you discuss asthma with your contacts?	What impact does your contact have on your asthma management?	How has your contact influenced your asthma management?
**Level of influence questions.**	How important is your contact to your asthma management?	Why do you feel your contact has such an impact on your asthma management?	What determines how important your contact is to your asthma management?

ALTER	Quote
GP	*" It's usually just to get a repeat prescription." ID41*
	[The preferred GPs] in the practice, you've actually got to plan to be sick. You've got to wait for an appointment like four weeks in advance.' ID10
	'Specialist is difficult but not impossible. GPs and all that, I've never had trouble getting in to see a GP or a pharmacist."ID13
	It's scary to think that as a patient you know more than they[GPs] do.'……<br>"They're either very good or dreadful. I had one tell me once I wasn't asthmatic because I had never been admitted to hospital with it." ID10
	" It was actually my GP who brought it up because she suffers from asthma and hay fever and she said she's noticed when she gets a hay fever attack her asthma is aggravated I guess." ID23
Respiratory Specialist	*"I trust [the respiratory specialist] with my life. He has helped me enormously over the years and I don't even really trust the GPs." ID27*

	'Well I don't see the specialist as often, I'd make it number 2.'ID2<br>*'I go to the specialist about asthma to find out if there's anything new for asthma.'ID2*
	" We're doing the spirometry and we're doing all the other things but we never really talk about hayfever." ID23
Allergist	*"Time's pretty precious and you just think well are they going to give me something constructive to help? Or maybe I should go see a naturopath and see.? Then people suggest the desensitization with your allergies and that."ID10*
Media	*"There was some interest a while about with kids and asthma I heard on the news and I did a bit of reading about that."ID10*
	"Probably the asthma foundation, their booklets I would I have seen at work." ID12
Friends and Colleagues	*"Other sufferers because I value their opinion-what they do. I don't think they are as important as a doctor. They'll probably lead you to somebody else to talk to." ID13*<br><br>*'The carer's support group is good for the stress, because stress affects your immune system and your asthma.' ID3*

Nurses	*You just go see her [no appointment required]. If she wants you to change your medications then you just make an appointment with the doctor and you get all that sorted out.'ID10*<br>*"The asthma educator is high on my list because she knows what she's doing." ID10*<br>*"That was good[education]. Probably a two." ID36*
ENT	*'The ENT is an allergy specialist. He's put me on permanent antihistamines'. ID3*
Pharmacist	*'I think when I found out I didn't need to see the GP anymore, I've just been to the pharmacist.' …..'They ask me how my asthma's going and whether or not I'm on any other medication for it but they do-I'm sure I've been asked do I need to see a specialist or have I been to the doctor's?'ID55*<br><br>*"They showed me how to administer like Symbicort® when I first had it. I didn't know how to use it and they showed me how to do that as well"……*<br>*"She guides me how many puffs to use and when is the right time to use it, and when I use the puffs, how to hold my breath and for how long. With this I was never taught anywhere*

	else."ID16
INTERNET	*'I was checking about the correct way to take medication. With the inhaler thing." ID21*<br>*'Sometimes I print it off and take it with me to my GP and discuss it. So it's sort of like I try and do it with them rather than apply it on my own because I know that things online are not always accurate" ID23*
ALTERNATIVE THERAPIST	*"For me, [breathing exercises] wasn't good. I ended up having to go back on medication." ID21*
PHYSIOTHERAPIST	*"how to take the puffer and things like that and to use a spacer always use a spacer."ID12*<br>*'they were doing chest clearance because my asthma is quite severe.' ID3*<br>*'I'd seen a physio…she does asthma education as well.' ID10*
DERMATOLOGIST	*'With prednisone I get a lot of haemorrhaging, bad haemorrahaging under the skin. He does check-I'm on hirudoid every day.' ID3*
OPTHALMOLOGIST	*"Because of all the cortisone I'm on it's affecting glaucoma and cataracts. I've got cataracts. They're not advanced enough. He said it*

	always carries a risk."ID3
AMBULANCE	*"They had to call the ambulance out to me to take me up to A and E"ID10*
